# Clinical and Genetic Profiles in Chinese Patients with Huntington’s Disease: A Ten-year Multicenter Study in China

**DOI:** 10.14336/AD.2018.0911

**Published:** 2019-10-01

**Authors:** Hong-Lei Li, Xiao-Yan Li, Yi Dong, Yan-Bin Zhang, Hong-Rong Cheng, Shi-Rui Gan, Zhi-Jun Liu, Wang Ni, Jean-Marc Burgunder, X. William Yang, Zhi-Ying Wu

**Affiliations:** ^1^Department of Neurology and Research Center of Neurology in Second Affiliated Hospital, and Key Laboratory of Medical Neurobiology of Zhejiang Province, Zhejiang University School of Medicine, Hangzhou, China.; ^2^Department of Neurology, First Affiliated Hospital, Fujian Medical University, Fuzhou, China.; ^3^Department of Neurology, Huashan Hospital, Shanghai Medical College, Fudan University, Shanghai, China.; ^4^Swiss Huntington’s Disease Centre, Siloah, Gümligen and, Department of Neurology, University of Bern, Bern, Switzerland.; ^5^Center for Neurobehavioral Genetics, Jane and Terry Semel Institute for Neuroscience and Human Behavior, Department of Psychiatry and Biobehavioral Sciences, Brain Research Institute, David Geffen School of Medicine, University of California at Los Angeles, CA, USA.; ^6^Joint Institute for Genetics and Genome Medicine between Zhejiang University and University of Toronto, Zhejiang University, Hangzhou, China

**Keywords:** Huntington’s disease, Chinese population, phenotype, genotype

## Abstract

Huntington’s disease (HD) is an autosomal dominant inherited neurodegenerative disorder caused by CAG triplet repeats expansion in exon 1 of the Huntingtin gene (*HTT*). In China, HD is considered to have a low prevalence. The goal of this study was to describe the clinical characteristic and genetic profiles of HD in a Chinese cohort. A total of 322 individuals with expanded CAG repeats were consecutively recruited from the neurologic clinics of three medical centers in Southeastern China between 2008 and 2018. Among them, 80 were pre-symptomatic mutation carriers and 242 were symptomatic patients. The mean age at onset (AAO), defined here as the age at motor symptom onset, of the 242 manifest HD individuals was 40.3 ± 11.9 years and the mean CAG repeat length was 46.1 ± 7.5 in the group of symptomatic patients. Initial symptoms were abnormal movements in 88.8% of the patients with psychiatric symptoms in 6.2%, cognitive impairment in 3.3% and others in 1.7%. The AAO of motor was negatively correlated with the CAG repeat length in an exponential regression analysis (R 2 = 0.74, P<0.001). Analysis of 46 parent-child pairs showed that the CAG repeat length was longer in the offspring group (45.8 ±7.6) than in the parent group (43.8 ±3.0) (p=0.005). Overall, this study provides clinical and genetic profiles in a cohort of Chinese patients with HD, which should contribute to a better understanding of this disorder.

Huntington’s disease (HD) is an autosomal dominant inherited neurodegenerative disorder characterized by movement disorder (i.e. chorea and dystonia), cognitive decline and psychiatric symptoms. HD is caused by CAG triplet repeats expansion in the exon 1 of the Huntingtin gene (*HTT*) [1]. Symptoms usually begin insidiously at mid-adult life, and inexorably worsen until patients’ premature death, usually in about 15-20 years after the motor symptom onset [2]. In general population, the normal* HTT* gene contains a sequence of 6-26 CAG repeats. Individuals who inherit an expanded CAG repeat of 40 or more will manifest the disease with a complete penetrance, whereas those who inherited 36-39 CAG repeats may be affected with milder penetrance. Although the intermediate alleles (27-35 CAG repeats) are considered not to be in a pathogenic range, they are unstable alleles and may expand or contract during the germline transmission [3].

HD prevalence is about 4-10 per 100,000 in the Caucasian populations but varies greatly between ethnic origins [4]. Among East Asians and South Africans, the prevalence of HD is reportedly to be only 0.1-1 per 100,000 [5-7]. Recently, an epidemiological study found the average annual incidence rate of HD in Taiwan China to be 0.1/100,000 [8]. In the absence of a nationwide epidemiological study in mainland China, the prevalence of HD in the Han Chinese population remains unknown. Further, there is a paucity of clinical phenotype description in this population.

In this study, we aim to provide a description of the clinical characteristics, genetic profile in a cohort of Han Chinese HD patients and gene carriers. We compared our findings with those reported in other ethnic populations around the world. These data are of great significance in guiding the clinical practice, especially in practical diagnostic process and genetic counselling. Moreover, the clinical and genetic studies of HD in different ethnic populations may provide new insights into this disorder.

## MATERIALS AND METHODS

### Subjects

A total of 322 individuals with expanded triplet repeats (80 pre-symptomatic mutation carriers and 242 manifest HD) from 238 families were consecutively recruited from the neurologic clinics of three medical centers (Second Affiliated Hospital of Zhejiang University School of Medicine, First Affiliated Hospital of Fujian Medical University and Huashan Hospital affiliated to Fudan University) in China between February 2008 and March 2018. HD diagnosis was made by at least two senior neurologists according to patients’ clinical manifestations in the context of positive *HTT* genetic tests. Predictive tests were performed in relatives as they requested after appropriate genetic counsel. Pre-symptomatic mutation carriers refer to those who show no motor signs and no other subjective symptoms or clinical sign which would be typical for HD. Comprehensive demographic and clinical data, including medical and psychiatric history, HD family history, and current medications were collected from the participants. All participants signed the informed consent form, or if the participants were unable to provide consent, an authorized representative provided the consents on their behalf. The study was approved by local Ethics Committees (i.e. Institutional Review Board) for medical research at three participating hospitals.

The age at onset (AAO) is defined by the age when motor signs or symptoms occur. Homozygous HD gene carriers refers to individuals with two expanded alleles.

### Genetic analysis of the HTT gene

Genomic DNA was extracted from peripheral blood treated by ethylene diamine tetraacetic acid (EDTA). Genetic testing for* HTT* gene was performed using the method previously reported [9]. Briefly, the primers of *HTT* (F: 5′ -CAGAGCCCCATT CATTGCC-3′ and R: 5′-TGAGGAAGCTGAGGA GGC-3′) were used to amplify the exon 1 in the CAG polymorphic region of the* HTT* gene. Polymerase chain reaction (PCR) amplicons were electrophoresed on a 1.5% agarose gel at 180V for twenty minutes to determine the primary *HTT* results. Then the length of CAG repeats was determined by Sanger sequencing.

### Statistical analyses

Demographic, clinical characteristics and genetic profile were analyzed using descriptive statistics. The data distributions of AAO and CAG were assessed by Kolmogorov-Smirnov test. AAO was normally distributed yet the CAG was distributed with a skew. For comparison between two continuous variables, T tests, Wilcoxon tests or Mann-Whitney were used as appropriate. ANOVA was used for comparison between multi-group. Correlation was calculated by Pearson or Fisher exact Chi-Square when appropriate. Statistical analysis was conducted using SPSS 20.0. The p-value less than 0.05 was considered statistically significant.

**Table 1:** The profile of persons with HD in our cohort.

	Number
Patients	322
Manifest HD	242
Premanifest HD	80
Family	238
Clear transmit mode	178
Paternal/ Maternal	95/83
Uncertain family history	34
Unknown family history	26
CAG available	309

**Figure 1.**
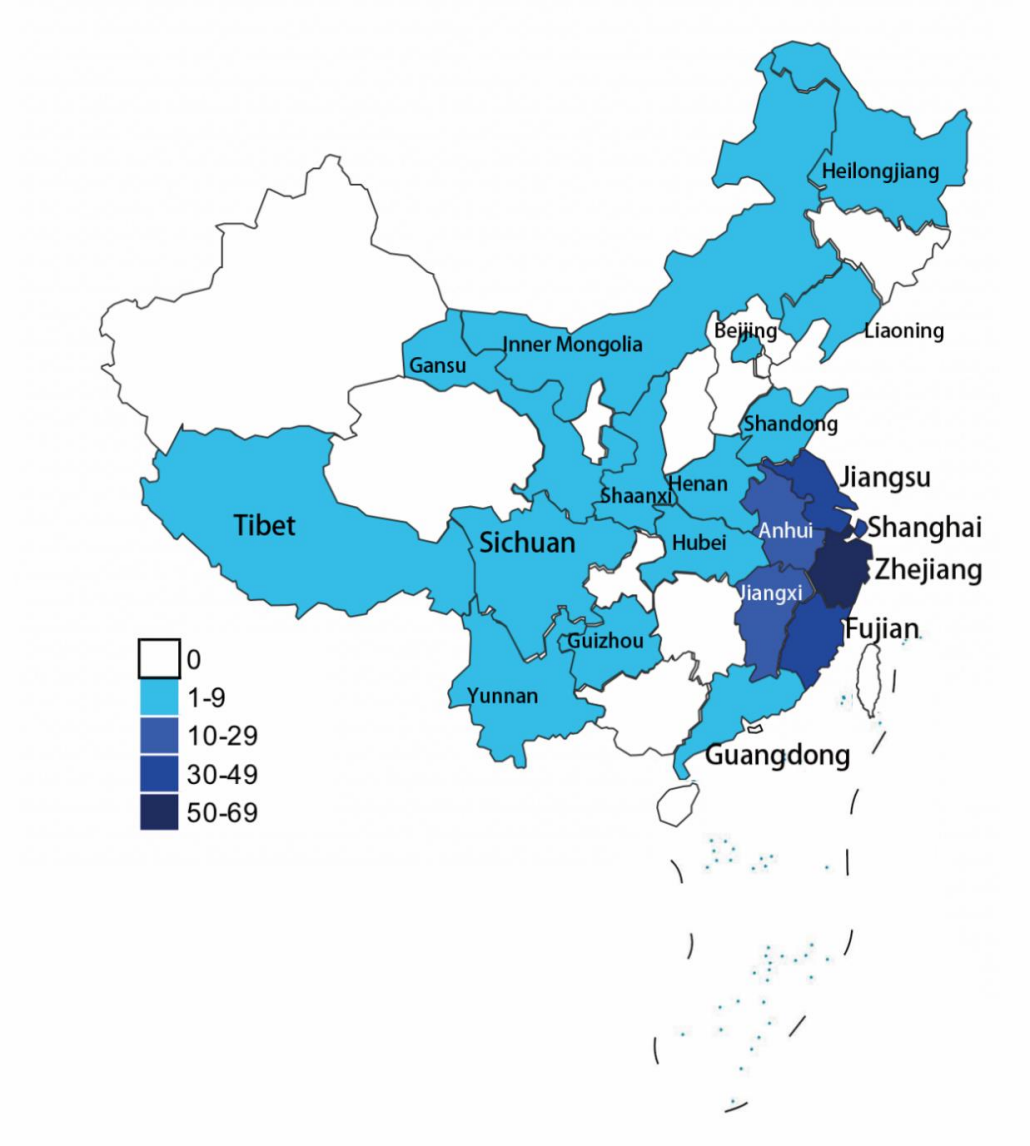
The geographical distribution of HD patients. Most patients were from Southeast China.

## RESULTS

### Population features

As shown in Table 1, among the 322 persons with abnormal repeats from 238 families, 242 (131 males and 111 females) were symptomatic patients while 80 (35 males and 45 females) were pre-symptomatic mutation carriers. To control against bias created by the presence of several family members, the index case from each family was included to calculate the transmission mode. Among 238 families, 178 had clear transmission mode (95 paternal and 83 maternal inheritance) and 34 denied their parents had any known chorea-like movements and information from the remaining 26 was lacking. Data on CAG repeat numbers was obtained from 309 subjects. DNA was missing from the remaining 13 persons. The mean age at genetic test was 29.9 ± 11.7 years (range 1-66 years) in the pre-symptomatic carriers with their mean number of CAG repeats of 44.2 ± 4.0 (range 37-59). The majority of the families were from Southeastern China (Fig. 1). The Unified Huntington’s Disease Rating Scale (UHDRS) was applied to assess the motor, cognitive and psychiatric symptoms. Age at onset was assessed in a structured interview and by use of all clinical reports. Assessments were performed by trained neurologists.

**Table 2:** Age at motor onset and CAG repeats among juvenile HD, adult HD and elder HD.

	AAO (years)		CAG		Age at diagnosis (years)		DOD (years)	

	Mean (SD)	Range	Mean (SD)	Range	Mean (SD)	Range	Mean (SD)	Range
Total (n=242)	40.3 (11.9)	4-71	46.1 (7.5)	38-104	45.7 (12.4)	7-83	5.4 (4.3)	0.25-30
male	39.7 (12.8)	8-71	46.5 (8.0)	39-104	45.1 (13.4)	10-83	5.5 (4.4)	0.25-27
female	41.0 (11.0)	4-60	45.6 (6.9)	38-92	46.4 (11.2)	7-68	5.4 (4.2)	0.25-30
Juvenile HD (n=15)	14.5 (4.8)	4-20	67.0 (16.8)	46-104	20.9 (9.3)	7-42	6.4 (6.9)	1-27
male	14.8 (4.2)	8-20	65.8 (18.6)	46-104	21.2 (10.2)	10-42	6.4 (8.3)	1-27
female	14.0 (6.2)	4-19	69.0 (14.8)	56-92	20.4 (8.3)	7-28	6.4 (3.3)	3-10
Adult HD (n=220)	41.4 (9.4)	21-59	44.9 (3.6)	39-58	46.7 (10.1)	23-68	5.3 (4.1)	0.25-30
male	40.7 (9.8)	21-59	45.2 (3.5)	39-58	46.1 (10.6)	23-68	5.4 (3.9)	0.25-23
female	42.0 (9.1)	21-59	44.6 (3.4)	40-54	47.3 (9.5)	26-68	5.3 (4.3)	0.25-30
Elderly-onset HD (n=7)	63.7 (4.8)	60-71	39.3 (0.9)	38-41	69.6 (6.7)	63-83	5.8 (3.2)	3-12
male	63.7 (4.9)	60-71	39.3 (0.9)	38-41	70.8 (7.7)	63-83	5.6 (3.8)	3-12
female	60	60	38.5	38-39	66.5	65-68	6.5	5-8

DOD: Duration from onset to diagnosis

### Clinical spectrum

#### Age at onset

**Figure 2.**
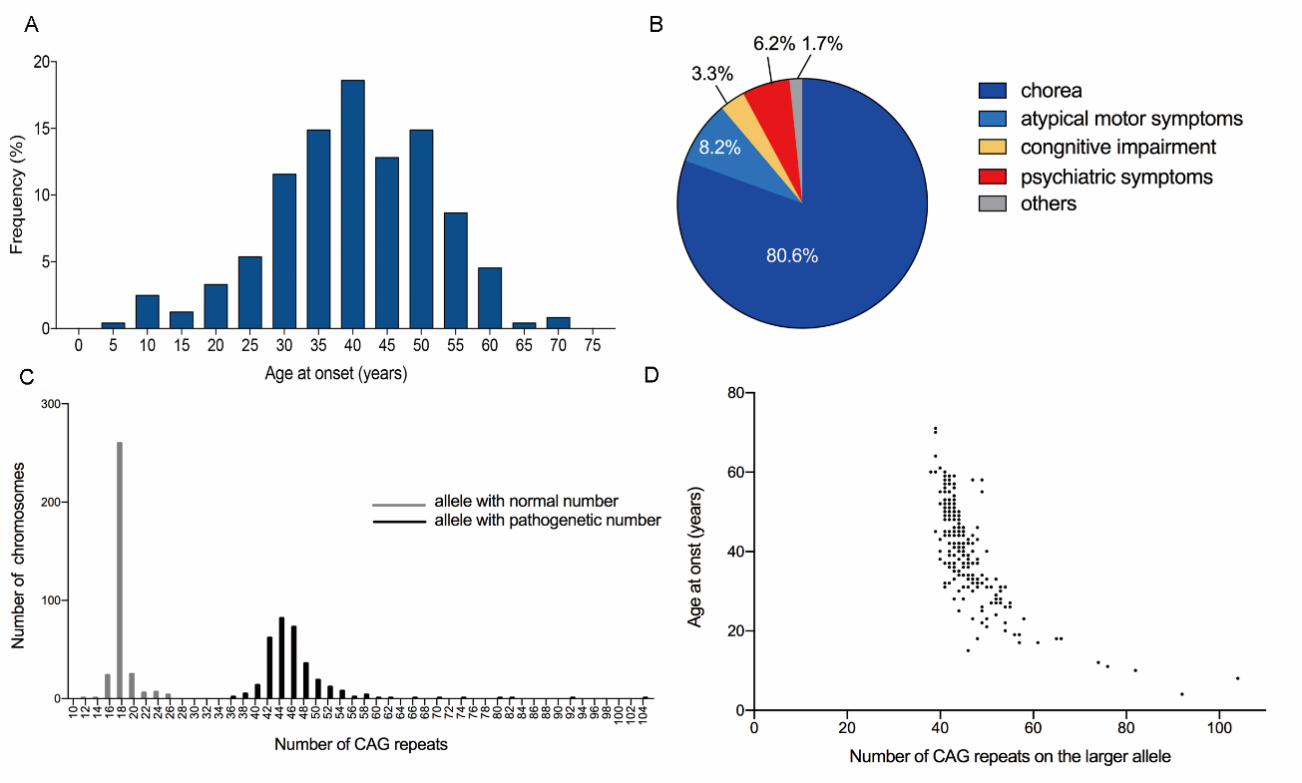
Clinical and genetic features of Chinese patients with HD. A) Frequency distribution of age at onset (AAO) in Chinese HD patients (n=242). The AAO occurred mainly on 40 years of age. B) Spectrum of manifest HD with initial symptom (n=242). Chorea was the most frequent initial symptom in manifest HD, accounting for 80.6%. The others were atypical motor symptom and cognitive or psychiatric symptom. C) Frequency distribution of CAG repeats number with normal and expanded range in affected individuals with HD (n=306). D) Relationship between AAO and number of CAG repeats on the larger allele of *HTT* gene (n=242). The CAG repeats could explain approximately 74% of variance in AAO.

Reliable data on AAO were available in 242 HD patients. The mean motor AAO was 40.3 ± 11.9 (range 4-71) years (Table 2) (Fig. 2A). The average age at diagnosis was 45.7 ± 12.4 (range 7-83) years with an average diagnostic delay of 5.4 years. Fifteen (6.2%) had a juvenile onset with a mean AAO of 14.5 ± 4.8 (range 7-20) years, and 7 (2.9%) had an old age onset with a mean AAO of 63.7 ± 4.8 (range 60-71) years. The other 220 patients (90.9%) had adult onset with an average mean AAO of 41.4 ±9.4 (range 21-59) years. (Table 2).

#### Spectrum of initial symptoms

Among the 242 symptomatic gene carriers, 215 first presented with motor signs (88.8%, 117 males and 98 females) and 27 (11.2%, 14 males and 13 females) presented with non-motor symptoms and developed motor disorder later. Chorea was the most frequent motor sign (n=195, 80.6%). Other types of motor symptoms included ataxia (n=7, 2.9%), gait instability (n=9, 3.7%), parkinsonism (n=1, 0.4%), tics (n=1, 0.4%), tremors (n=1, 0.4%) and dystonia (n=1, 0.4%). Eight patients (3.3%, 7 males and 1 females) had cognitive impairment first, of whom 5 with memory decline and 3 with intellectual decline. Fifteen subjects (6.2%, 4 males and 11 females) had psychiatric and behavioral symptoms, including agitation (n=9, 3.7%), depression (n=5, 2.1%) and psychosis (n=1, 0.4%). In addition, two patients had mixed psychiatric and cognitive impairment at onset and one patient had concurrent movement disorder and cognitive decline. Finally, one juvenile patient presented with seizure as initial symptom (Fig. 2B). Gender had a significant different distribution in motor symptoms group vs psychiatric symptom group (p=0.04), or cognitive symptom group vs psychiatric symptom group (p=0.01) as females had a higher frequency in psychiatric symptom group compared with the other groups. However, there was no difference for gender between motor and cognitive groups (p=0.08).

#### Duration of disease

Data of 97 deceased HD patients from 238 families were analyzed to estimate the duration of disease. Seven of them died by accident (n=2), suicide (n=2), cerebral hemorrhage (n=2) or because of esophageal cancer (n=1, at the age of 62). Hence, these seven patients were not included in the further analysis of disease duration.

As shown in Table S1, the mean duration of disease was 13.3 ± 6.3 (range 6-53) years defined by the mean AAO (42.9 ± 10.4 years, range 21-70 years) and mean age at death (56.2 ± 11.1 years, range 38-83 years). The median duration of disease was 11 years. To examine whether duration of disease was related to AAO, we divided participants into three groups: 20-39 years, 40-59 years and 60 years and older. There was no difference between these three groups (p=0.50) with a mean disease duration of 14.1 ± 8.6, 13.3 ± 5.0 and 11.6 ± 2.1 years, respectively. There was no effect of gender on duration of survival (p=0.71).

### Atypical HD patients

#### Juvenile HD(JHD)

Fifteen had juvenile onset (mean 14.5 ± 4.8 years, range 4- 20 years) with a mean CAG value of 67.0 repeats (Table S2). Four cases had been reported previously [10]. The initial symptoms of these patients were variable, including gait instability (n=4), chorea (n=4), intellectual decline (n=2), tics (n=1), tremor (n=1), depression (n=1) and dystonia (n=1), paroxysmal seizure (n=1). Three cases had no family history. Inheritance was paternal in 9 cases (75%) and maternal in 3 (25.0%). Diagnostic delay in JHD patients was 6.4±6.9 (range 1-27) years (Table 2).

#### Elder HD

Seven patients developed symptoms at the age of 60 years or older (mean 63.7 ± 4.8 years, range 60-71 years) (Table S3). Six had chorea as their initial symptoms and the other one had depression five years prior to the onset of chorea. Nearly all elder HD patients complained of memory decline, some also had dysarthria and irritability. Four (66.7%) had paternal and 2 (33.3%) maternal inheritance, whereas one had unknown family history. Expanded CAG triplet number was relatively small (mean 39.3 ± 0.9, range 38-41) (Table 2). Diagnostic delay was 5.8 ± 3.2 years.

#### Homozygous HD

Three homozygotes for elongated CAG repeats were found in our cohort (Table S4). One was reported in 2012 as first homozygous HD (asymptomatic individual) in China with CAG repeats of 37/42 [11]. Two additional cases were subsequently found with CAG repeats of 36/47 and 37/56, respectively. The reported female (CAG= 37/42) [11] was still asymptomatic at 48 years of age (in 2018). The second patient with 37/56 CAG repeats suffered from developmental delay and intellectual decline starting at 14 years old. Five years later, she developed chorea and gait instability. The third one (CAG=36/47) first presented with chorea at the age of 40 years. Afterwards, he developed memory decline at 44 years, and irritability, dysarthria and dysphagia 4 years later. For the three homozygotes, the mean CAG repeat length of the longer allele was 48.3 ± 7.1, ranging from 42 to 56 repeats. The mean AAO and diagnostic delay was 29.5 ± 14.8 years and 6.5 ± 4.9 years (n=2), respectively.

**Table 3:** Median AAO for each number of CAG repeats in Chinese patients and Dutch patients.

CAG Repeats	Chinese cohorts (n=232)			Dutch cohort (n=755)		
	No. of patients	Median AAO (y)	95%CI	No. of patients	Median AAO (y)	95% CI
<=40	13	60	59-62	21	68	55-81
41	19	50	45-53	28	65	58-72
42	32	50.5	47-52	90	64	60-68
43	32	49.5	44-49	80	60	56-64
44	27	42	40-44	101	55	52-58
45	22	39.5	37-41	104	50	47-53
46	17	38	33-40	100	45	43-47
47	14	35.5	32-42	63	41	39-43
48	13	33	31-39	50	37	35-39
49	9	32	26-45	34	38	36-40
50	5	31	20-39	19	34	30-38
51	2	29	4-54	8	33	27-39
52	5	28	22-34	11	29	19-29
>=53	22	19.5	16-23	46	23	20-26

CI: Confidence Interval. Y: years

### Distribution and instability features of CAG repeats

Data on CAG repeat numbers were available for 309 persons with HD. The three homozygotes mentioned above were excluded. The range of triplet repeats in the normal allele was 11-26 (mean 17.6 ± 1.6) while in the expanded allele, the range was 37-104 (mean 45.6 ± 6.9) (Fig. 2C).

To analyze the CAG instability during the germline transmission, we selected 46 parent-child pairs from our cohort, including 23 father-child pairs from 23 families and 23 mother-child pairs from 20 families (as one mother had three children). The gender of the affected parent was an important factor affecting the CAG repeats instability. The mean CAG repeat number was 43.4 ± 3.3 in father group and 46.4 ± 10.4 in corresponding child group, expanding by 3.0 ± 7.9 repeats during father-child transmission. In mother-child transmission, the CAG expanded by 0.9 ± 2.6 repeats with the CAG repeat number of 44.3 ± 2.7 in the mother group and 45.2 ± 3.2 in the corresponding child group. There was a difference in the father-child transmission (p=0.014) but not in the mother-child transmission (p=0.145). By adding together, the CAG repeat length was more significantly longer in the offspring group (45.8 ± 7.6) than the parent group (43.8 ± 3.0) (p=0.005).

As shown in Table S5, on HD chromosomes, intergenerational CAG changes occurred in 71.7% (33/46) of meiosis, with contraction accounting for 23.9% (11/46) and expansion for 47.8% (22/46). In the remaining 28.3% (13/46) intergenerational transmissions, the CAG number was unchanged. Among 22 expanded transmissions, only two (9.1%) transmission expanded greater than 7 repeats and they were both paternal inheritances. The expansion of CAG repeats ranged from 1 to 37 whereas contraction ranged from -3 to -1. Paternal transmitted mutant *HTT* alleles showed similar proportion compared with the maternal transmitted ones.

**Table 4:** The number of CAG repeats, AAO and initial symptoms among different countries.

Genetic and clinical features		Mainland	Thai [14]	Dutch [12]	Korean [17]	Mexican [16]	African [6]
Expanded CAG	Mean	46.1	43.5	46.0	45.4	47.2	44.5
	SD	7.5	3.0	4.0	4.7	5.4	4.0
	Range	38-104	39-48	39-71	40-58	37-106	37-57
P value		/	0.15	0.80	0.58	0.01*	0.21
AAO (years)	Mean	40.3	37.8	47.0	46.5	37.4	N
	SD	11.9	8.3	15.0	12.7	12.9	N
	Range	4-71	27-58	4-86	24-69	2-78	N
P value		/	0.38	<0.001*	0.004*	0.002*	/
Initial symptom	Motor	88.8%	N	N	89%#	53.0%	N
	Behavior	6.2%	N	N	36%#	30.0%	N
	Cognition	3.3%	N	N	28%#	3.5%	N
Cohort number		242	18	614	36	691	37

N: means the article didn't provide.

* P value was calculated when compared our cohort data with other countries’.

# The article of Korea counted initial symptom by first visit

### Correlations between CAG, AAO and initial symptoms

Correlations between the AAO, CAG and initial symptoms were assessed in 242 cases. To explore whether CAG repeat length and AAO differed with respect to initial symptoms, we divided patients into motor, cognitive and psychiatric groups. The CAG repeat number was larger in cognitive group (52.8 ± 11.6) than motor group (46.0 ± 7.4) (p=0.03) and psychiatric group (44.4 ± 6.3) (p=0.02). Accordingly, the AAO of cognitive group (25.1 ± 9.1 years) was earliest compared with motor group (40.5 ± 11.8 years) (p=0.0003) and psychiatric group (40.2 ± 11.1 years) (p=0.0035). This suggested that patients presented with cognitive impairment first had larger CAG number and earlier AAO. Further, we found chorea become more predominant with the increasement of AAO, from 26.7% (4/15) in JHD, 83.6% (184 in 220) in adult HD to 85.7% (6 in 7) in elder HD.

The AAO was inversely correlated with the expanded CAG repeat number (p<0.0001), and the CAG repeat lengths accounted for up to 74% of the variance in AAO (r= -0.86, R^2^ = 0.74) using the exponential regression analysis (Fig. 2D). We then separately tested their correlation in juvenile HD, adult HD and elder HD, and the R^2^ was 0.63, 0.49, and 0.05, respectively. Strong associations were detected both in juvenile HD and adult HD (both p< 0.001). However, no correlation was found between CAG repeats and AAO in elder HD (p = 0.31). This indicated that the AAO of elder HD was not affected by the CAG repeat lengths.

To predict the AAO more accurately, we divided patients into groups according to the number of CAG repeats and calculated each median AAO. We then compared our result with that of Dutch population (Table 3) [12]. Median AAO in Chinese population was earlier than that in Dutch population by each number of CAG repeats.

## DISCUSSION

Previous studies have suggested that the length of CAG repeats in normal HTT chromosomes is associated with the prevalence of HD in distinct populations. In a population with higher prevalence of HD, the size of normal CAG repeats was generally larger [13]. Our study showed that the mean CAG repeats of normal allele was 17.6 ± 1.6 (range 11-26), which is lower than in the European population (18.4 ± 3.7, range 8-35, n=479, p<0.001) [14]. Of note, normal alleles and intermediate alleles in the present study are limited to those determined in clinical samples of HD, which may result in an ascertainment bias. In our previous study, 483 healthy Chinese controls were sequenced in the *HTT* gene region and the mean CAG repeat number was 18.9 ± 2.57 (range 9-35) as well as the frequency of intermediate alleles was 2.5% (24/966) [15].

The size of CAG triplet on expanded chromosomes was within a range of 38 to 104 (mean 46.1 ± 7.5) and the mean AAO was 40.3 ± 11.9 (range 4-71) years (Table 4). Mexicans [16] had longer CAG length (47.2 ± 5.4, n=691) than in our cohort. Accordingly, they had earlier AAO (37.4 ± 12.9 years) (p=0.002). However, Korean and Dutch population had similar CAG length (45.4 ± 4.7, n=36 [17]; 46.0 ± 4.0, n=614 [12]) to our cohort (p=0.6, 0.8, respectively), but their AAO was later than in our cohort with Korean population (46.5 ± 12.7 years) (p=0.004) and Dutch population (47.0 ± 15.0 years) (p<0.001). This may be caused by genetic differences with distinct modifiers present in one ethnic population but not in the other. The comparison of CAG length and AAO is shown in Table 4.

The negative correlation between CAG repeat length and AAO has been found in many studies in different populations and confirmed here again [12, 16]. In our cohort, 74% of the variation for AAO could be explained by the CAG repeat number, which is higher compared with a Belgian cohort (50.4%) [18] and a Venezuelan cohort (72%) [19]. This indicates that other genetic and environmental factors also contribute to the remaining variation of AAO. A study on the monozygotic twins also suggested that different living conditions and individual habits might influence AAO [20].

We then analyzed various onset symptoms and found that the symptoms were quite diverse in juvenile onset HD. However, in adult HD and elder HD, the onset symptoms were relatively simple, usually including chorea-like symptoms. This suggests that larger CAG repeats (generally >60) cause initial symptoms of great variety. Movement symptoms were the predominant initial symptoms among Chinese HD patients (89%), Korean patients (89%) [17], American (69%) [21] and Mexican (53%) [16]. Notably, there were only 23 (9.5%) patients whose initial symptoms were cognitive decline or psychiatric symptoms in our study. This was much lower than previously reported, estimated at approximately 33% in patients with Mexican origin [16].

In our cohort median duration of disease was 11 years, which is shorter than in the Leiden Roster, in which median survival in 800 HD patients was 16.2 (range 2-45) years from motor symptom onset [2]. Furthermore, disease duration was independent of AAO, which is confirmed in our analysis. Another cohort containing 4,448 DNA samples and 848 detailed disease cases with duration data also showed that the CAG repeats number determined age at death rather than disease duration [22]. Unexpectedly, our median disease duration was far less than reported cases in Europe and North America. On the one hand, it may be caused by the relatively low standard of living condition and lack of access to health-care services in the past. On the other hand, this difference may be related to uncertainties in AAO ascertainment.

Frequency of juvenile onset HD cases (AAO <21 years) and homozygous HD in other studies are 4%-10%[23-25] and 0.1%-0.4% [26], respectively. In our study, the corresponding frequencies were 6.2% (15/242) and 0.9% (3/322), which is in a similar range. The lack of family history in a subgroup of our cohort, designated to uncertain family history, may be due to earlier death of the parents [27].

In summary, this study presents the clinical characteristics and genetic profile of HD in the Han Chinese population. This constitutes a solid foundation for future clinical, genetic and therapeutic investigation of HD in China. However, there are some limitations of this study. First, the participants were recruited from three centers located at the Southeastern China, thus there might be some population bias. Second, because of the low prevalence of HD in China, many physicians or even general neurologists lack the knowledge for accurate diagnosis. As a result, the diagnosis of many patients is delayed, and the prevalence of HD and the duration of disease is likely to be underestimated. Further studies with additional patients are needed for a more comprehensive and accurate epidemiological survey of HD in Mainland China.

## Supplementary Materials

The Supplemenantry data can be found online at: www.aginganddisease.org/EN/10.14336/AD.2018.0911
