# Supplementary material for: Clinical and Genetic Profiles in Chinese Patients with Huntington’s Disease: A Ten-year Multicenter Study in China
Source: Aging Dis. 2019 Oct 1;10(5):1003–11. doi: 10.14336/AD.2018.0911 (PMC6764736; doi:10.14336/AD.2018.0911)
Supplement: Supplementary file 1 — The Supplemenantry data can be found online at: www.aginganddisease.org/EN/10.14336/AD.2018.0911 [file AD-10-5-1003-s.pdf]

SUPPLEMENTARY DATA

Clinical and Genetic Profiles in Chinese Patients with Huntington's Disease: A Ten-year Multicenter Study in China

Hong-Lei Li[1#], Xiao-Yan Li[1#], Yi Dong[1#], Yan-Bin Zhang[2], Hong-Rong Cheng[1], Shi-Rui Gan[2], Zhi-Jun Liu[3], Wang Ni[1], Jean-Marc Burgunder[4&], X. William Yang[5], Zhi-Ying Wu[1, 6*]

[1]Department of Neurology and Research Center of Neurology in Second Affiliated Hospital, and Key Laboratory of Medical Neurobiology of Zhejiang Province, Zhejiang University School of Medicine, Hangzhou, China. [2]Department of Neurology, First Affiliated Hospital, Fujian Medical University, Fuzhou, China. [3]Department of Neurology, Huashan Hospital, Shanghai Medical College, Fudan University, Shanghai, China. [4]Swiss Huntington's Disease Centre, Siloah, Gümligen and, Department of Neurology, University of Bern, Bern, Switzerland. [5]Center for Neurobehavioral Genetics, Jane and Terry Semel Institute for Neuroscience and Human Behavior, Department of Psychiatry and Biobehavioral Sciences, Brain Research Institute, David Geffen School of Medicine, University of California at Los Angeles (UCLA), Los Angeles, USA. [6]Joint Institute for Genetics and Genome Medicine between Zhejiang University and University of Toronto, Zhejiang University, Hangzhou, China

© 2018. Li HL et al. Published online at http://www.aginganddisease.org/EN/10.14336/AD.2018.0911

SUPPLEMENTARY DATA

Supplemental Table 1. Duration of disease among 90 HD deceased patients.

	No. of patients	AAO (years)		Age at death (years)		DOD (years)		*p* value of
		**Mean (SD)**	**Range**	**Mean (SD)**	**Range**	**Mean (SD)**	**Range**	**duration**
Overall	90	42.9 (10.4)	21-70	56.2 (11.1)	38-83	13.3 (6.3)	6-53	
AAO								
20-39	32	32.7 (3.7)	21-39	46.9 (8.7)	38-83	14.1 (8.6)	6-53	
40-59	46	45.0 (5.5)	40-57	58.2 (8.2)	46-78	13.3 (5.0)	6-33	0.50
>59	12	61.7 (3.5)	60-70	73.2 (3.9)	70-81	11.6 (2.1)	10-16	
Sex effect								
Male	42	45.6 (11.7)	21-70	59.2 (12.8)	38-83	13.6 (7.8)	6-53	0.71
Female	48	40.5 (8.5)	30-60	53.6 (9.9)	40-73	13.1 (4.6)	6-26	

DOD: Duration from onset to diagnosis

Supplemental Table 2. Clinical features and number of CAG repeats in 15 juvenile HD cases.

Patient	AAO(y)/Gender	CAG	Initial symptoms/symptom onset age	DOD(y)	Symptoms developing	Affected parent
1	4/F	18/92	gait instability intellectual decline/4y	3	dysarthria, memory decline	Father
2	8/M	17/104	seizure, chorea/8y	2	gait instability, memory decline,11y died	Father
3	10/M	18/82	tics/10y	3	chorea, gait instability, dysarthria	Uncertain
4	11/M	17/76	intellectual decline, memory decline/9y	2	chorea, dysarthria, gait instability	Father
5	12/M	NA	gait instability, agitated/12y	1	dysarthria and intellectual decline	Father
6	12/F	17/74	gait instability, chorea/12y	7	dysarthria, dysphagia, bradykinesia	Father
7	15/M	17/46	chorea/15y	27	memory decline, anxiety	Uncertain
8	17/F	17/57	gait instability 17y	9	chorea/26y, psychiatric disorder, agitation	Mother
9	17/M	18/61	chorea/17y	3	chorea	Father
10	18/F	22/66	tremors/18y	10	psychiatric symptoms/26y, dysarthria/27y and dysphagia, gait instability/28y	Father
11	18/M	17/65	depression/10y	2	obsession/18y, chorea	Father
12	18/M	17/48	chorea/18y	15	chorea	Uncertain
13	19/F	37/56	developmental delay and intellectual decline/14y	3	chorea/19y, gait instability/20y	Father
14	19/M	17/57	dystonia/19y	2	apathy/20y	Mother
15	20/M	17/54	chorea/20y	7	dysarthria, memory decline, psychiatric symptoms	Mother

DOD: duration from onset to diagnosis; F: female; M: male; y: years

Supplemental Table 3. Clinical features and number of CAG repeats in seven elder HD cases.

© 2018. Li HL et al. Published online at http://www.aginganddisease.org/EN/10.14336/AD.2018.0911

SUPPLEMENTARY DATA

Patient	AAO(y)/Gender	CAG	Initial symptoms/onset age	DOD(y)	Symptoms developing	Affected parent
16	60/F	17/39	chorea/60y	8	memory decline	Mather
17	60/M	17/41	chorea/60y	3	memory decline	Father
18	60/F	17/38	depression/55y	5	chorea, gait instability, memory decline	Father
19	61/M	16/40	chorea/61y	6	memory decline, dysarthria, irritability	Mather
20	64/M	17/39	chorea/64y	4	dysarthria, walking instability	Father
21	70/M	17/39	chorea/70y	3	memory decline	Father
22	71/M	17/39	chorea/71y	12	memory decline, dysarthria, irritability	UA
					dysarthria, irritability, sleep disorder	

DOD: duration from onset to diagnosis. UA: unavailable; F: female; M: male; y: years.

Supplemental Table 4. Clinical features and number of CAG repeats in three homozygotes HD cases.

Patient	AAO(y)/Gender	CAG	Initial symptoms/onset age	DOD(y)	Symptoms developing	Affected parent
13	19/F	37/56	developmental delay and intellectual decline/14y	3	19y chorea, 20 gait instability	Father
22	now 48/F	37/42	carrier	None	None	Mother
23	40/M	36/47	chorea/40y	10	44y memory decline	Mother
					48y irritability, dysphagia, dysarthria	

DOD: duration from onset to diagnosis; F: female; M: male; y: years

Supplemental Table 5. The influence of CAG sizes and transmission mode on intergenerational CAG instability.

	Parent CAG	Offspring CAG	CAG change	Paternal/maternal	P value of ΔCAG
Total pairs(n=46)				23/23	0.005
Mean (SD)	43.8 (3.0)	45.8 (7.6)	1.9 (5.9)		
Range	39-55	39-92	-3-37		
Total Expansion (n=22)				13/9	0.005
Mean (SD)	43.8 (3.4)	48.9 (10.0)	5 (7.4)		
Range	39-55	42-92	1-37		
Expansions>7repeats (n=2)				2/0	0.35
Mean (SD)	48.5 (4.9)	71.5(29.0)	23.0 (19.8)		
Range	42-55	51-92	9-37		
Expansions =<7 repeats(n=20)				9/11	0.0001

© 2018. Li HL et al. Published online at http://www.aginganddisease.org/EN/10.14336/AD.2018.0911

Mean (SD)	43.4 (2.4)	46.6 (2.9)	3.2 (1.8)		
Range	39-48	42-52	1-7		
No change (n=13)				5/8	1.00
Mean (SD)	43 (2.2)	43 (2.2)	0		
Range	40-47	40-47	0		
Contractions (n=11)				5/6	0.0002
Mean (SD)	44.8 (3.0)	43 (2.3)	-1.8 (1.1)		
Range	40-49	39- 46	-3-0		

© 2018. Li HL et al. Published online at http://www.aginganddisease.org/EN/10.14336/AD.2018.0911
